# Rapid Estimation of Binding Activity of Influenza Virus Hemagglutinin to Human and Avian Receptors

**DOI:** 10.1371/journal.pone.0018664

**Published:** 2011-04-13

**Authors:** Yang Cao, Xiaoying Koh, Libo Dong, Xiangjun Du, Aiping Wu, Xilai Ding, Hongyu Deng, Yuelong Shu, Jianzhu Chen, Taijiao Jiang

**Affiliations:** 1 National Laboratory of Biomacromolecules, Institute of Biophysics, Chinese Academy of Sciences, Beijing, China; 2 Graduate University of Chinese Academy of Sciences, Beijing, China; 3 Department of Biological Engineering, Massachusetts Institute of Technology, Cambridge, Massachusetts, United States of America; 4 State Key Laboratory for Molecular Virology and Genetic Engineering, National Institute for Viral Infectious Disease Control and Prevention, Chinese Center for Disease Control and Prevention, Beijing, China; 5 Center for Infection and Immunity, Institute of Biophysics, Chinese Academy of Sciences, Beijing, China; 6 Department of Biology, Koch Institute for Integrative Cancer Research, Massachusetts Institute of Technology, Cambridge, Massachusetts, United States of America; Kyushu Institute of Technology, Japan

## Abstract

A critical step for avian influenza viruses to infect human hosts and cause epidemics or pandemics is acquisition of the ability of the viral hemagglutinin (HA) to bind to human receptors. However, current global influenza surveillance does not monitor HA binding specificity due to a lack of rapid and reliable assays. Here we report a computational method that uses an effective scoring function to quantify HA-receptor binding activities with high accuracy and speed. Application of this method reveals receptor specificity changes and its temporal relationship with antigenicity changes during the evolution of human H3N2 viruses. The method predicts that two amino acid differences at 222 and 225 between HAs of A/Fujian/411/02 and A/Panama/2007/99 viruses account for their differences in binding to both avian and human receptors; this prediction was verified experimentally. The new computational method could provide an urgently needed tool for rapid and large-scale analysis of HA receptor specificities for global influenza surveillance.

## Introduction

The first critical step in influenza virus infection and transmission is binding of the viral surface protein, hemagglutinin (HA), to receptors on host cells. The ability of HA to bind terminal sialic acids that have different linkages with the penultimate galactose unit determines whether a virus can infect birds or humans or both (host tropism). The natural hosts of influenza viruses are aquatic birds, which predominantly express α2–3 sialylated glycans; an avian virus has to gain the ability to bind human receptors, which are α2–6 sialylated glycans, in order to cross the species barrier and infect humans [1], [2], [3]. In recent years, the avian influenza virus H5N1 has been a major public health concern because of its ability to cause a high rate of mortality in infected individuals and an increasing incidence of human infections. In the event that the H5N1 virus gains the ability to bind well to human receptors, it might acquire the capacity for easy human-to-human transmission and cause an influenza pandemic.

Knowing the receptor specificity of HA is also critical for the timely production of influenza vaccines, because the most widely used method for vaccine production requires growing human influenza viruses in embryonated chicken eggs. If the HA of the human virus does not bind well to receptors in chicken eggs, vaccine production could be adversely affected. For example, the World Health Organization (WHO) recommended the use of influenza A/Fujian/411/02 (H3N2) virus for vaccine production for the 2003–2004 flu season. However, the A/Fujian/411/02 virus failed to replicate well in chicken eggs [4], [5], possibly due to weak HA binding to sialic acid receptors in the allantoic and amniotic cavities. As a result, an earlier H3N2 strain of A/Panama/2007/99 had to be used in its place for vaccine production. The antigenic difference between the two strains was so large that immunity induced by A/Panama/2007/99 did not protect against infection by the Fujian-like viruses, rendering the vaccine ineffective during the 2003–2004 flu season [6], [7]. Using reverse genetics technology, it has been shown that two amino acid changes of either G186V and V226I, or H183L and V226A are sufficient for the Fujian virus to adapt for growth in eggs [4]. Differences in amino acids at positions 155 and 156 account for the antigenic differences between the Panama and Fujian viruses [5]. Despite these progresses, little is known about the molecular basis for the altered receptor binding specificity in the evolution of Fujian-like viruses from the A/Panama/2007/99 virus, or the evolutionary relationship between changes in antigenicity and receptor binding specificity.

Given that the receptor binding specificity of HA directly affects influenza transmission from avian species to humans, it is imperative to develop robust and effective methods for monitoring changes in the receptor binding specificity of influenza viruses as they evolve. Major efforts have been devoted to understand the molecular mechanisms governing HA binding specificities by determining crystal structures of HA-receptor complexes and analyzing HA-glycan binding using glycan arrays [8], [9], [10], [11], [12], [13], [14], [15], [16], [17]. Computational models have also been developed to assess HA-receptor binding on a small scale. Several studies have used the ab initio fragment molecular orbital method, in conjunction with molecular dynamics and molecular mechanics approaches, to calculate HA-receptor binding activity [18], [19], [20], [21], [22], [23], [24]. However, the utility of these computational methods is limited because they lack systematic validation and tend to be computationally expensive, which poses a barrier for practical applications in influenza surveillance.

Here, we report a novel computational method that uses an effective scoring function to quantify the binding strength of HA-receptor interactions with high accuracy and speed. We demonstrate the utility of this method, with a focused large-scale sequencing and experimental verification, in identifying molecular events that underlie the receptor specificity changes during the evolution of human H3N2 Fujian-like viruses.

## Results

### A rapid computational method for predicting HA-receptor binding activity

The binding of an influenza virus to a host cell can be approximated by the interaction between HA and the host receptor analog, sialyloligosaccharides [25]. In this study, the α2–3 pentasaccharide (LSTa, Neu5Acα(2–3)Galβ(1–3)GlcNAcβ(1–3)Galβ(1–4)Glc) and α2–6 pentasaccharide (LSTc, Neu5Acα(2–6)Galβ(1–4)GlcNAcβ(1–3)Galβ(1–4)Glc) were used for avian and human receptor analogs, respectively. Previously, Xu et al found that when binding to HAs, the pentasccharide receptor analogs, seemingly quite flexible, exhibit limited binding modes [23]. We also found that binding positions of the receptor analogs relative to HAs are highly conserved and the backbone conformation at receptor binding region is almost fixed, when analyzing the co-crystal structures involving HAs from different type A viruses. Therefore, to propose a computational framework to rapidly estimate the binding strength of HA and the receptor analogs, we used the predominant topologies of cone-like and umbrella-like ones to represent avian and human host receptor analog structures, respectively [26], and further fixed their relative binding positions as observed in the co-crystal structures involving the same subtype of HA (see Materials and Methods). Such an approximation allows us to identify the comparative impacts of different HA sequences on receptor binding while keeping other factors consistent. Our computational method consists of four steps (details see Materials and Methods). First, for an HA of interest, its structure was constructed based on homology modelling using an available HA crystal structure that is highly similar to the target HA (>70% sequence identity). Second, the HA structure was aligned against known HA-receptor complex templates and the binding position of the receptor analog in the HA receptor binding domain was initialized. Third, the conformations of side chains at receptor binding sites were refined. Fourth, the binding strength of HA and receptor analog was evaluated by an effective empirical scoring function developed in this study (see below). The computations took less than 2 minutes on an Intel Xeon 2.8 GHz processor, much less computationally demanding than traditional molecular dynamics techniques that usually took weeks for binding free energy calculations. Thus, the approach developed here can be used in large-scale applications such as the rapid monitoring of evolution of influenza receptor binding specificities.

### An effective scoring function for evaluating HA-receptor binding strength

The critical step in our computational method was to develop a scoring function that can quantify the binding strength between an HA and avian/human receptor anlog. To this end, we developed an empirical scoring function by taking into account the effect of electrostatics (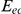) and shape complementarity (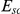) on HA-receptor binding, described as below: 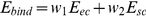
(1)


where the 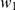 and 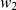 are the weights. The electrostatic interaction term, 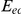, uses the inter-molecular Coulomb term: 
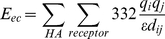
(2)


where *d_ij_* is the distance of HA atom *i* and receptor atom *j*. And the *q_i_* and *q_j_* are the atom charges. 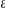 is the dielectric constant which is set to be 1.

Shape complementarity is a geometric descriptor for delineating the geometric match at the binding interface between interacting molecules. Usually it is based on either molecular surface curvatures or surface areas. Here, we propose a curvature weighted surface area model to calculate the shape complementarity term, 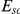. We first follow the classic method of Connolly ML [27] to quantify the surface curvature by using a probe sphere centering at the solvent accessible surface (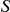) and calculating the ratio of sphere volume out of the solvent accessible surface (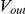) to its total volume (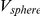). Then, a shape score 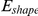 is calculated by integrating the surface curvature over the solvent accessible surface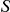:
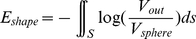
(3)


In this formula, a logarithm operation is introduced to enhance the sensitivity to cavities on surface. Finally, the shape complementarity term 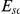 considers the buried shape score as a result of binding, which is calculated as follows:
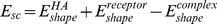
(4)


Since our scoring function can be generalized to score other molecular interactions, we first trained to fit the predicted binding scores to the measured on PDBbind database [28], a set of protein-ligand complexes with crystal structures and experimentally determined *K*_i_ and *K*_d_ values. In the training, we attempted to obtain highest Pearson's correlation coefficients between the predicted binding scores and the measured *K*_i_ or *K*_d_ values. We found it has a desirable performance (see Table S1), which is comparable to the best existing scoring functions of same kind (see Table S2). To further improve its performance in the prediction of HA-receptor binding activity, the equation (1) was retrained on the experimental data consisting of apparent association constants for binding of 21 HAs with avian/human receptor analogs [12] (See Table S2). For each of the 21 HAs, we calculated the differed values of electrostatic term and shape complementarity term between α2–6 receptor and α2–3 receptor:
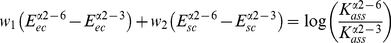
(5)


The parameters *w*_1_ and *w*_2_ were obtained by linear regression analysis of the above equation using least squares. The calculated binding scores have a relatively good correlation with the experimental data (*w*_1_ = −0.05 and *w*_2_ = 0.057, Pearson's correlation coefficient  = 0.67, P value  = 0.0009, and standard deviation  = 0.90). Based on this training method, 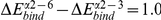 represents about 10 times more binding preference for human receptor than for avian receptor.

### Predicted critical mutations on HAs correlate with experimental measurements

Recent efforts on characterizing the effects of amino acid mutations on HA-receptor binding specificity have provided experimental data with which we can assess the performance of our computational method. The amino acid substitutions that were experimentally identified to change the binding strength to either avian or human receptor, especially the receptor preference were regarded as critical mutations. Several well-characterized critical mutations for HAs of H1N1, H3N2 and H5N1 viruses were collected from literature (Table S3). To validate whether our predictions are consistent with the experimental observations, for each of these mutations, the receptor binding strength for the interactions of HA with avian and human receptors were computed both for the wild type and mutated HA to yield the change 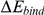:
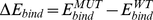
(6)


To achieve a best correlation with the experimental data, a single mutation on HA that causes a change in binding strength with an absolute value of 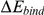 ≥1.0 when binding to either avian or human receptor was regarded as a predicted critical mutation (≤−1.0: decreased binding activity (↓), and ≥1.0: increased binding activity (↑)) (Table S3). Based on these criteria, our method successfully predicted the well studied critical mutations that are responsible for receptor binding activities, including residue 190 and 225 (H3 numbering) for H1N1 [29], residue 226 and 228 for H3 subtypes [30], [31] and residue 186 and 196 (H3 numbering) for H5N1 [17]. Overall, 19 of 22 (∼86%) predicted critical mutations matched those observed in experiments, demonstrating the reliability of our method in quantifying effect of HA mutations on their binding to avian/human receptors.

### Predicted receptor binding preferences of influenza A viruses correlate with host origins

**Figure 1:**
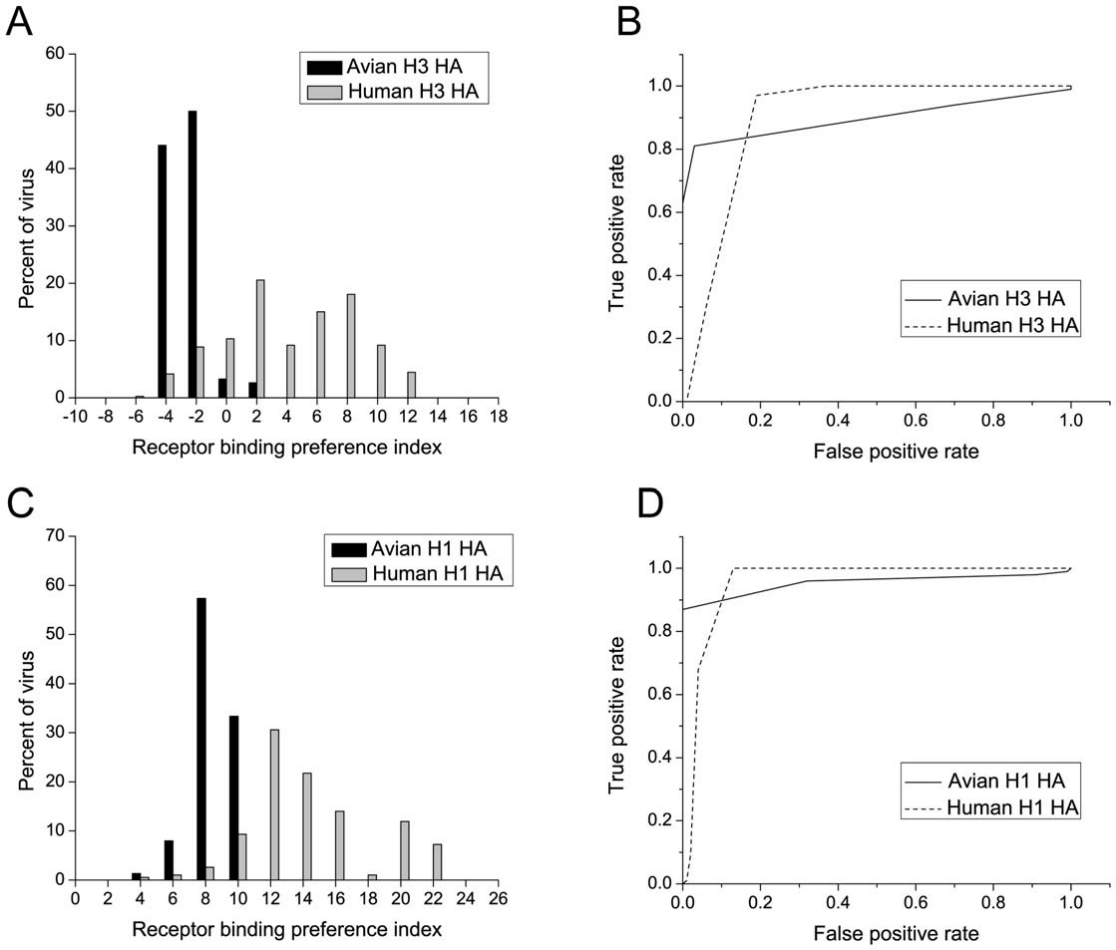
Predicting receptor binding preferences of natural H1, H3 and H5 viruses. A, C, The distribution of H3 (A) H1 (C) viruses isolated in humans and avian species according to their receptor binding preference indices are defined as the difference of binding score to the human and avian receptor analogs (
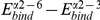
). 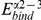 and 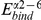 indicate the predicted binding strength of HA with the avian receptor analog LSTa) and the human receptor analog (LSTc), respectively. B, D, Receiver-operator characteristic (ROC) curves of predicting human/avian H3 viruses (B), human/avian H1 viruses (D). The ROC curves are plotted as rate of true positives as a function of rate of false positives at different values of 
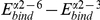
.

Next, we determined whether the computational method was able to discriminate between avian viruses and human viruses based on the predicted receptor binding preferences. For this purpose, we collected all the H1 and H3 avian and human viruses and the avian H5N1 viruses including those infected humans up to 2008 from the NCBI Influenza Virus Resource. After removing the redundant sequences, 193 human H1N1 viruses, 75 avian H1N1 viruses, 360 human H3N2 viruses, 152 avian H3N2 viruses, 686 avian H5N1 viruses and 144 human-infecting H5N1 viruses were selected (see Methods S1). Given that the human and avian receptor analogs may not represent their respective actual receptors equally well, we did not directly compare their calculated binding strengths to the same HA. To compare their binding preference, we used a receptor binding preference index, which was quantified as the difference between the binding strengths for the human receptor and that for the avian receptor (
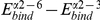
). A larger value indicates a greater preference for human receptors and vice versa. In the case of H3N2 viruses, the average receptor binding preference index for avian H3N2 viruses is −1.5, considerably smaller than that for human H3N2 viruses, which is 5.2. Fig. 1A shows that the distribution of avian and human H3N2 HAs according to the predicted receptor binding preference index. At a cut-off of 0.0,86.7% human H3N2 viruses and 94.1% avian H3N2 viruses show their natural receptor preferences respectively. Fig. 1C shows that the computational method also distinguished human and avian H1N1 viruses based on the predicted receptor binding preference index: the average value for avian viruses is 9.5, much smaller than 15.2 for human viruses. At a cut-off of 12.0,86.5% human and 100% avian viruses show their host receptor preferences respectively.

We quantified the performance of the computational prediction by constructing receiver-operator characteristic (ROC) curves. In ROC curves, the true positive rate (Y-axis) was plotted as a function of the false positive rate (X-axis) for different cut-off values, and thus the closer is the curve to upper left corner, the better the prediction performance is. As shown in Fig. 1B and 1D, the ROC plots of both H3 and H1 tests are close to the upper left corner, indicating that the computational method is effective in distinguishing avian and human viruses based on their HA receptor binding strengths. These analyses show that predicted receptor binding preferences are highly correlated with the host specificities of natural influenza A viruses.

In the case of H5N1 viruses, although avian H5N1 viruses have started to infect humans sporadically, the human-infecting H5N1 viruses have not been adapted to humans and cannot be efficiently transmitted between humans, indicating their binding specificities have not been changed. Notably, in our calculation, we also found human-infecting H5N1 viruses and other avian H5N1 viruses are indistinguishable in the predicted receptor binding specificities (Fig. S1A, S1B), which is consistent with the fact that the human-infecting H5N1 viruses have not been adapted to humans. This suggests that our calculation is valid.

### Application of the computational method to track the evolution of receptor binding specificities of human H3N2 viruses

**Figure 2:**
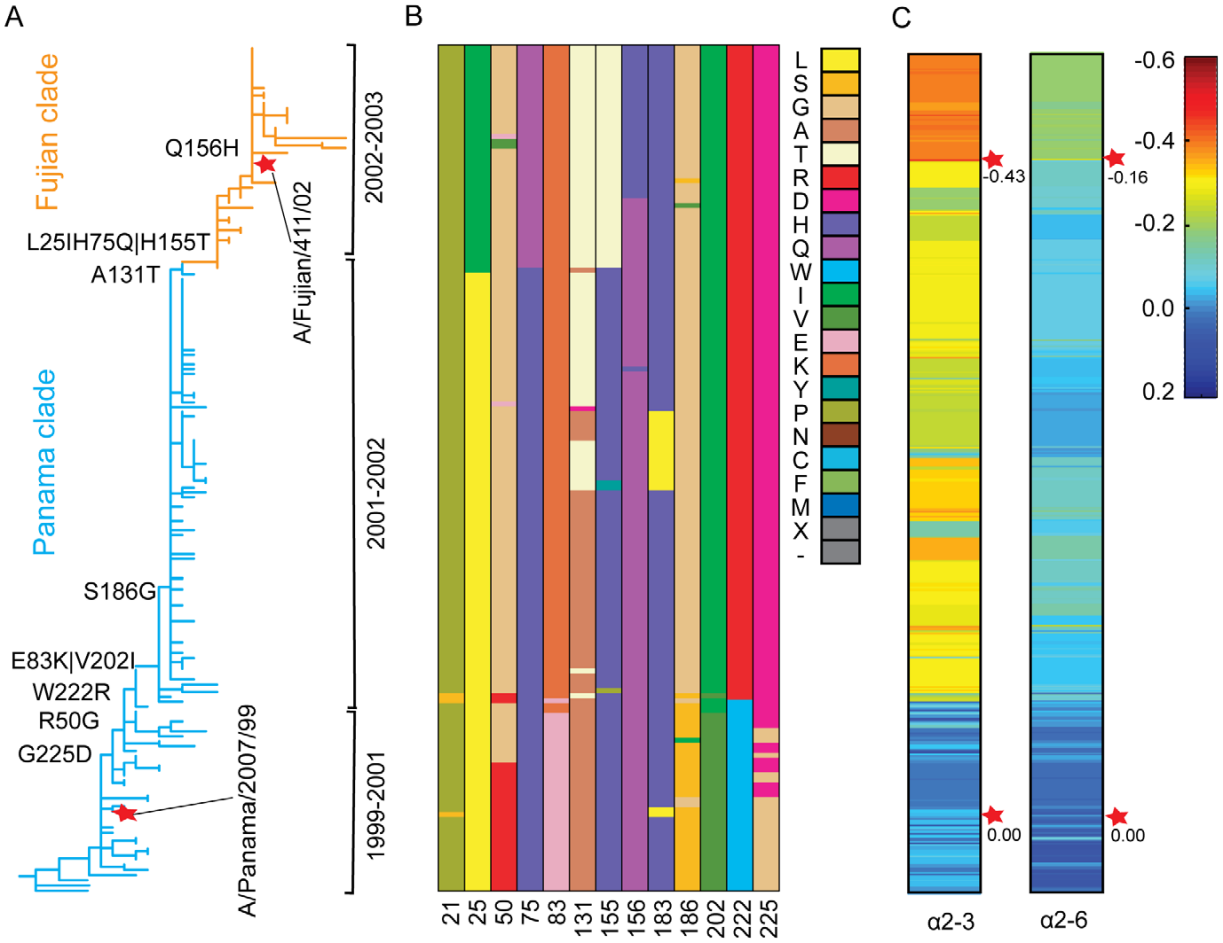
Evolution of receptor binding specificity of the Panama- and Fujian-like viruses. A, Phylogenetic analysis of evolutionary history of human H3N2 viruses isolated in China from year 2000 to 2002 covering flu seasons from 1999–2000 to 2002–2003. The red stars denote the position of A/Panama/2007/1999 virus and A/Fujian/411/2002 virus on the phylogenetic tree. Key amino acid changes are shown at the indicted positions during the evolution from Panama-like viruses to Fujian-like viruses. B, Dynamic changes of amino acids at 13 sites that differ between Panama-like viruses and Fujian-like viruses alined along the phylogenetic tree in A. Color code of amino acids, represented by a single letter, is shown. ‘X’ and ‘-‘ indicates unknown amino acid and gaps, respectively in the HA sequence. C, Comparison of calculated binding strengths for all HAs in A to both avian (α2–3) and human (α2–6) receptor analogs, normalized to those of HA of A/Panama/2001/99 by 
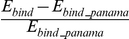
. The heat map of binding strength is aligned with the corresponding HAs in the phylogenetic tree to indicate dynamic change of the binding strengths of these viral HAs to both avian and human receptors during evolution. Scale of normalized binding strength values is shown. From year 1999 to 2003, cool colours changed to be hot colours. It reflects the binding strength was decreased, especially for the avian receptor analog. The red stars denote the normalized binding strength values of A/Panama/2007/1999 virus and A/Fujian/411/2002 virus.

To test the value of the computational method for influenza surveillance, we determined the molecular events underlying receptor specificity changes in the evolution of the Fujian-like (H3N2) viruses by combining the binding strength predictions with large-scale HA sequencing. HA genes from a total of 207 human H3N2 viruses isolated from diverse regions in China between 2000 and 2002 were sequenced. Based on these sequences, phylogenetic analyses of these H3N2 HAs revealed two temporally distinct clades that we designate as Panama and Fujian, after the WHO-recommended vaccine strains of the A/Panama/2007/99 and A/Fujian/411/02 viruses (Fig. 2 and Fig. S2). The Fujian clade appeared in China in the 2002–2003 flu season, one season earlier than in the United States (Fig. S2A, S2B). To gain molecular insights into the evolution of the Fujian clade, we further tracked the detailed amino acid changes along the phylogenetic tree (Fig. 2B). As shown in Fig. 2B, the focused sequencing effort uncovered remarkable sequence diversity present in the HA1 subunit, allowing a visual representation of almost all amino acid substitution intermediates between the Panama-like viruses and the Fujian-like viruses over time.

To uncover the receptor binding changes during the evolution of the Fujian-like viruses, the binding strengths of HA to both the human and avian receptor analogs were calculated for each of the 207 virus isolates. Fig. 2C shows the dynamic change of binding strength of HA to both the human and avian receptor analogs during evolution by tracking the binding strengths for each HA on the phylogenetic tree (Fig. 2A). It is noticeable that the decrease in binding strengths for both the avian receptor and human receptor occurred twice during the evolution of Fujian-like viruses from Panama-like viruses: the first one occurred at the beginning of the 2001–2002 flu season and the second one at the beginning of the 2002–2003 flu season. A side-by-side, visual comparison of amino acid changes (Fig. 2B) and receptor binding changes (Fig. 2C) allows association of specific amino acid change(s) with alterations in receptor binding properties. For example, the mutations of W222R and G225D located at the receptor binding region took place at the beginning of the 2001–2002 flu season, when predicted binding strength decreased. By modelling the effect on receptor binding of each mutation at all thirteen sites that differed between A/Panama/2007/99 and A/Fujian/411/02 viruses (Fig. S3 and Fig. S4), we found that the mutations at residues 222 and 225 played the most important role in mediating receptor binding alterations in the Fujian-like viruses (The detailed analysis is given in the legends of Fig. S3).

### Experimental validation of the predicted molecular mechanism

**Figure 3:**
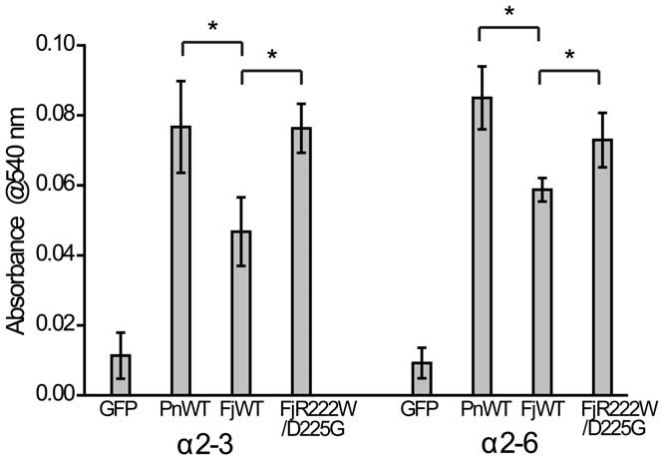
Experimental validation of amino acid residues critical for altered receptor-binding specificity of Fujian-like viruses. Comparison of receptor binding activities of wild type Panama and Fujian HAs and Fujian HA with R222W and D225G mutations (FjR222W/D225G). Briefly, the wild type and mutant HAs were expressed on the surface of 293T cells. Sialic acid was removed from chicken red blood cells with neuraminidase and resialylated to express either α2–3 or α2–6 linked sialic acid. The amount of red blood cells bound to HA expressed on the 293T cell surface was measured by absorbance at 540 nm. GFP-transfected 293T cells were used as a control for nonspecific binding. Representative data from one of the five experiments are shown. *p<0.05.

To test whether the amino acid changes at residues 222 and 225 considerably changed HA's receptor binding specificity, we mutated the residues 222 and 225 in HA of A/Fujian/411/02 virus back to the corresponding residues of HA of A/Panama/2007/99 viruses and measured binding of the double mutant and two wildtype HAs with either α2–3 or α2–6 linked sialic acid receptors by a hemadsorption assay (see Methods S1). In agreement with prediction, the wild type A/Fujian/411/02 HA exhibited a considerably weaker binding to both α2–3 and α2–6 receptors than the wildtype A/Panama/2007/99 HA (Fig. 3, p<0.05). When residues 222 and 225 in A/Fujian/411/02 HA were mutated to the corresponding residues of A/Panama/2007/99 HA, the resulting HA had a considerable increase in binding activity to both α2–3 and α2–6 receptors. These results confirm the prediction, showing that the changes in HA residues 222 (W to R) and 225 (G to D) indeed caused a considerably low receptor binding activity for α2–3 sialic acid receptor during the evolution of Fujian-like viruses.

## Discussion

Here, we report a novel computational method for measuring interaction strength between influenza HA and their host cell receptors. This method can predict binding strengths of a wide variety of influenza HA, and was rigorously tested for accuracy. Application of this computational method has enabled us to identify how receptor specificities change during the evolution of human H3N2 Fujian-like viruses. We predicted and further validated by experimentation that W222R and G225D mutations in HA result in a change in receptor binding specificity during the evolution of the Fujian-like viruses.

The computational method employs an effective scoring function which translates both sequence and structural information of HA into quantitative HA-receptor binding strength by evaluating the effects of electrostatic and shape complementarity. These two physical features have been widely used in studying protein-ligand interactions. They enable us to identify how binding strengths change with aspect to the amino acid changes. In this study, they are used to identify the critical mutations, changes in viral host-specificity, and binding activity changes during the course of virus evolution.

One major difficulty in computational modelling of HA-receptor binding is accurate representation of the receptors. Although sialyltrisaccharides or sialyldisaccharides were used in previous modelling [18], [19], [21], [22], [32], recent studies show that the human receptor moiety beyond the third glycan also interacts with HAs and plays a role in virus binding to human receptors [33]. In our study, we also used the shorter sialyltrisaccharide glycans and found that they did not discriminate receptor-binding specificity between avian and human influenza A viruses as well as the longer sialylpentasaccharide glycans. Thus, we used sialylpentasaccharide glycans in all the analyses. Although more accurate, the pentasaccharide receptor is still a simple representation of the physiological receptors and the prediction of receptor specificity of influenza virus is not straightforward. It is difficult to determine whether a virus binds preferentially to human or avian receptors by directly comparing its binding scores to the two different receptor ligands. To circumvent this problem, we interpret the binding strength or specificity comparatively among different HAs or compare relative binding strength or specificity to a reference HA. This interpretation can cancel out, to some degree, the unknown effects of various factors in comparison.

Accurate and timely monitoring of the evolution of HA's receptor specificity is critical for global preparedness for influenza epidemics and pandemics. Since their introduction into humans, the receptor-binding specificity of human H3N2 viruses has changed continuously [12], [34], [35], [36], [37], [38]. The 2003–2004 flu season was especially severe, because an effective vaccine against the highly virulent H3N2 Fujian-like strains was not available in time due to the poor growth of the Fujian-like virus in embryonated chicken eggs. Several groups have investigated the molecular basis underlying the poor replication of the Fujian-like viruses in embryonated chicken eggs. Using reverse genetics, Lu *et al.* found that the unbalanced HA receptor-binding activity and NA enzymatic activity in the Fujian virus contributes to its poor growth in embryonated chicken eggs. Better virus growth can be achieved by either increasing the HA receptor-binding activity via G186V and V226I mutations in HA or lowering the NA enzymatic activity via E119Q and Q136K mutations in NA [4]. By passaging the Fujian-like viruses in chicken eggs, it was found that mutations in HA not in NA improve the replication of the virus in chicken eggs and these mutations also increase HA binding activities to the avian receptors. Three different pairs of mutations, including G186V and V226I, H183L and V226A, or H183L and D188Y, have been identified in egg-adaptation of Fujian virus [4], [39], [40]. However, these mutations are unlikely to mediate the evolution of Fujian-like viruses in nature since none of them occurred in either Fujian clade or Panama clade.

By large scale sequencing of HAs of human H3N2 viruses sampled from different regions in China during 2000-2002 and computational modelling, we were able to trace the molecular events and characterize the evolutionary dynamics of receptor specificity changes in the evolution of Fujian-like viruses. We predicted that W222R and G225D mutations in HA result in a decreased binding activity to both human and avian receptors, particularly to avian receptors, during the evolution of the Fujian-like viruses. To test whether the decreased HA-receptor binding activity was accompanied by a decrease in viral infectivity in chicken eggs when the residues 222 and 225 were changed in the Panama clade, we compared the replication efficiency in chicken eggs of the viruses bearing different amino acids at residues 222 and 225 within the same antigenic Panama clade (see Methods S1). Wild type A/Panama/2007/99 viruses bearing 222W and 225G in HA replicated efficiently in chicken eggs, while the A/Panama/2007/99 viruses with a single mutation (222W or 225D) or double mutations (222R and 225D) replicated poorly (Fig. S5A, p = 0.0009; Fig. S5B, p = 0.057). The correlation between the decreased HA's receptor-binding activity and the poor viral growth in the chicken eggs suggests that the amino acid changes at residues 222 and 225 contribute to the poor replication of the wildtype A/Fujian/411/02 virus in chicken eggs.

Studies have shown that H155T and Q156H substitutions in HA were sufficient to render the Panama virus antigenically equivalent to A/Wyoming/03/03, an A/Fujian/411/02-like H3N2 virus [5]. By showing that W222R and G225D substitutions in HA mediate receptor specificity changes, we uncovered the evolutionary relationship between receptor specificity and antigenicity in the evolution of Fujian-like viruses. While antigenicity changes at residues 155 and 156 occurred in the 2002–2003 flu season, changes that impacted on receptor binding occurred over a year earlier at the beginning of the 2000–2001 flu season (Fig. 2A and 2C). It can be envisioned that such findings are of critical importance for global influenza surveillance as they can alert us earlier to prepare for changes in receptor-binding specificity and an imminent influenza epidemic or pandemic. Our approach can provide an urgently needed tool for rapid and large-scale analysis of HA receptor specificities for global influenza surveillance.

## Materials and Methods

### Computational method for predicting HA-receptor binding strength

Structure templates for HA-receptor complexes were obtained from Protein Data Bank(PDB) [41]. The HA moiety of 1RVX of H1N1 virus, 1MQN of H3N2 virus, and 2IBX of H5N1 virus were used as HA templates for their respective virus subtypes. Protein atom charges are obtained from CHARMM22 [42]. The avian and human receptor analogs were LSTa (Neu5Acα(2–3)Galβ(1–3)GlcNAcβ(1–3)Galβ(1–4)Glc) and LSTc (Neu5Acα(2–6)Galβ(1–4)GlcNAcβ(1–3)Galβ(1–4)Glc), respectively. As the complete structure of LSTa in complex with HA is not available, its coordinates were prepared from 2RFT and the glycosidic torsion angles were reset referring the modelling data by Xu *et al.* [23]. The coordinates of LSTc were extracted directly from 1RVT and 1JSI. Hydrogen atoms and charges were generated by the Dundee PRODRG2 Server [43].

The computational method to calculate HA-receptor binding strength consists of four steps. In step 1, the structure of the target HA sequence is built. The HA sequence is first aligned to the template HA of same subtype virus by CLUSTALW [44], then is threaded to the template, and finally the conformations of its side chains are modeled by a fast side chain construction program, SCWRL4 [45]. Any site with a gap or insertion is automatically ignored. In step 2, the receptor analog is transferred to the newly built HA in the same position as that relative to template HA in the template. Its coordinates were manually adjusted to avoid steric clashes with HA templates. In step 3, the amino acid side chains which contain atoms within a 12 Å distance from the receptor analog are repacked using a heuristic iteration search algorithm to optimize side chain conformations sequentially [46] based on an empirical scoring function parameterized over known HA structures (see Methods S1). This scoring function contains van der Waals, salt bridge and solvation effects. The side chain conformations use the Dunbrack rotamer library [47]. In step 4, protein hydrogen atoms are added according to their standard coordinates data from CHARMM22 [42]. Then HA-receptor binding strength is calculated according to a scoring function developed in our study (see text).

The software developed from the current study is free for non-commercial users at web site: http://jianglab.ibp.ac.cn/lims/harbp/harbp.html.

### Sequencing and sequence analysis

HA sequences used in this analysis were generated at the Chinese Influenza Center as part of an ongoing routine genetic analysis of HA genes of variant and typical influenza field strains. See Methods S1 for details.

### Hemadsorption glycan-binding assay

The hemadsorption glycan-binding assay protocol was modified from Glaser *et al.* [29]. See Methods S1 for details.

## Supporting Information

Figure S1
**Predicting receptor binding preferences of natural H5 viruses, related to** **Figure 1** **.** A, The distribution of H5 viruses isolated in humans and avian species according to their receptor binding preference indices are defined as the difference of binding score to the human and avian receptor analogs (
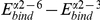
).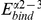 and 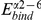 indicate the predicted binding strength of HA with the avian receptor analog LSTa) and the human receptor analog (LSTc), respectively. B, Receiver-operator characteristic (ROC) curves of predicting human-infecting/avian H5 viruses. The ROC curves are plotted as rate of true positives as a function of rate of false positives at different values of 
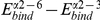
.(TIF)

Figure S2 **Tracking the evolution of receptor binding specificities of human H3N2 viruses, related to** **Figure 2** **.** (A-B) Phylogenetic analyses of the evolutionary histories of human H3N2 viruses isolated in China (A) and the USA (B) from 1999 to 2003. Phylogenetic tree analyses of 207 viruses isolated in China and 370 viruses isolated in USA from year 2000 to 2003 (covering flu seasons, 1999–2000, 2000–2001, 2001–2002 2002–2003, and 2003–2004). Color code of amino acids, represented by a single letter, is shown. ‘X’ and ‘–’ indicate unknown amino acids and gaps, respectively, in the sequences. Note: The amino acid changes usually occur in viruses isolated in China earlier than those isolated in the USA. H155T, Q156H, W222R and G225D indicate key mutational events in the evolution of the Panama clade to Fujian clade.
(TIF)

Figure S3 **Computational identification of amino acid residues critical for altered receptor-binding specificity of Fujian-like viruses, related to** **Figure 2** **.** A: 13 different residues (red) between Panama and Fujian viruses on the structure of HA1. The binding region is highlighted by a yellow circle. B and C, Comparison of calculated binding strength of wildtype Panama (Pn) and Fujian (Fj) HAs to avian (α2–3) (b) and human (α2–6) (c) receptor analogs with specific amino acid changes in the HAs. To pinpoint the molecular changes responsible for the receptor specificity changes in A/Fujian/411/02, we modeled the effect on receptor binding of each mutation at all thirteen sites that differed between A/Panama/2007/99 and A/Fujian/411/02 viruses. For binding to the α2–3 sialic acid receptor (B), most single amino acid changes on the A/Panama/2007/99 HA did not have much effect except for changes at four residues, 155, 186, 222 and 225, which resulted in significant decreases in binding strength compared to the wild type. When positions 222 and 225 were changed simultaneously, the binding strength was further decreased. For single amino acid changes on the A/Fujian/411/02 HA backbone, only two changes at residues 222 and 225 resulted in a significant increase in binding strength compared to the wildtype. Similarly, simultaneous changes at positions 222 and 225 resulted in further decrease in binding strength. For binding to the α2–6 sialic acid receptor (C), residue 222 and 225 stood out again in that change at this position in HA of A/Panama/2007/99 and A/Fujian/411/02 viruses exhibited the reciprocal effect. These calculations suggest that the mutations at residues 222 and 225, which occurred at the beginning of the 2000–2001 flu season, played the most important role in mediating receptor binding alterations in the Fujian-like viruses.
(TIF)

Figure S4 **HA1 sequence alignment of Panama and Fujian virus.** The 13 different residues are highlighted by the blue color. Receptor binding region comprises the 130-loop (134–142, H3 numbering), 150-loop (150–156), 190-helix (181–193) and 220-loop (220–230) (Yellow background).
(TIF)

Figure S5 **Comparison of growth of the Panama virus (222W/225G) and its variants 222W/225D and 222R/225D in embryonated chicken eggs, related to** **Figure 3** **.** A: Quantification by quantitative RT-PCR. The relative RNA copy is the ratio of the RNA copies in the embryonated chicken eggs after viral infection of 44 hours to those in the embryonated chicken eggs infected with same amount of viruses but kept frozen for 44 hours. (*p = 0.0009). B: Quantification by HA assay. The values of HA titers are average of either seven 222W/225G isolates, two 222W/225D isolates, or five 222R/225D isolates. (*p = 0.057)
(TIF)

Table S1 **Comparison with popular scoring functions on PDBbind database.**
(DOC)

Table S2 **Training data for the scoring function.**
(DOC)

Table S3 **Validation of the computational method using well characterized mutations.**
(DOC)

Methods S1 **Supporting methods.**
(DOC)
