# Supplementary figures and images for: Rapid Estimation of Binding Activity of Influenza Virus Hemagglutinin to Human and Avian Receptors
Source: PLoS One. 2011 Apr 13;6(4):e18664. doi: 10.1371/journal.pone.0018664 (PMC3076431; doi:10.1371/journal.pone.0018664)

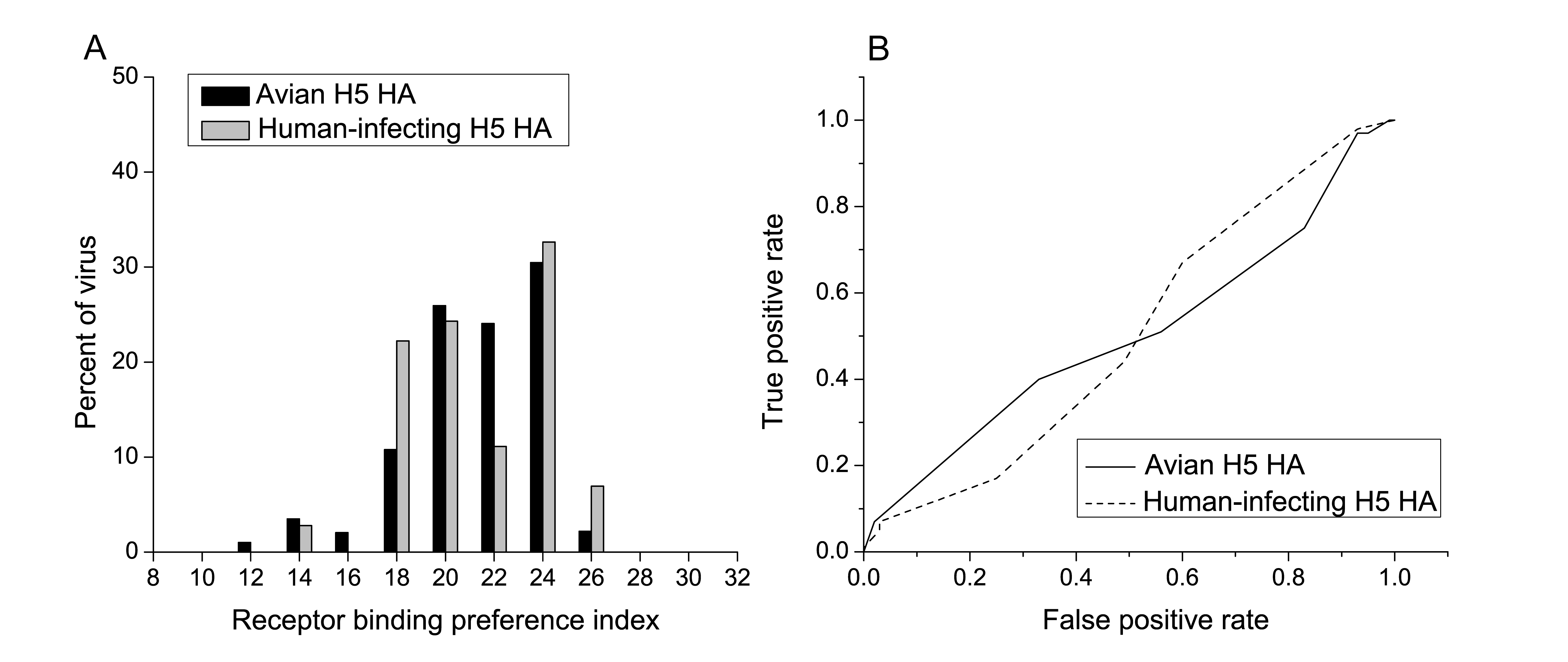

Supplement: Figure S1 — Predicting receptor binding preferences of natural H5 viruses, related to Figure 1 . A, The distribution of H5 viruses isolated in humans and avian species according to their receptor binding preference indices are defined as the difference of binding score to the human and avian receptor analogs (). and indicate the predicted binding strength of HA with the avian receptor analog LSTa) and the human receptor analog (LSTc), respectively. B, Receiver-operator characteristic (ROC) curves of predicting human-infecting/avian H5 viruses. The ROC curves are plotted as rate of true positives as a function of rate of false positives at different values of . (TIF) [file pone.0018664.s001.tif]

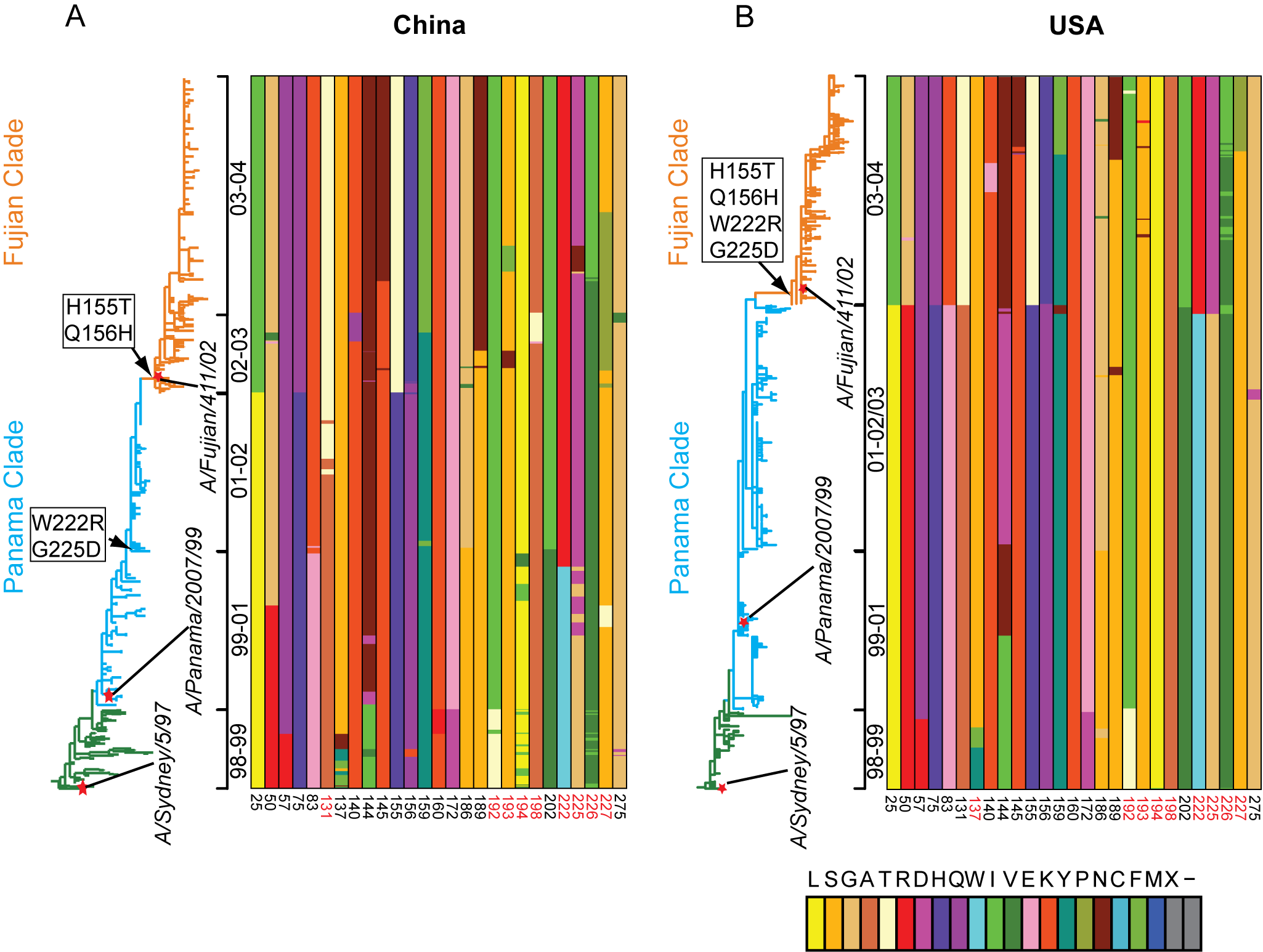

Supplement: Figure S2 — Tracking the evolution of receptor binding specificities of human H3N2 viruses, related to Figure 2 . (A-B) Phylogenetic analyses of the evolutionary histories of human H3N2 viruses isolated in China (A) and the USA (B) from 1999 to 2003. Phylogenetic tree analyses of 207 viruses isolated in China and 370 viruses isolated in USA from year 2000 to 2003 (covering flu seasons, 1999–2000, 2000–2001, 2001–2002 2002–2003, and 2003–2004). Color code of amino acids, represented by a single letter, is shown. ‘X’ and ‘–’ indicate unknown amino acids and gaps, respectively, in the sequences. Note: The amino acid changes usually occur in viruses isolated in China earlier than those isolated in the USA. H155T, Q156H, W222R and G225D indicate key mutational events in the evolution of the Panama clade to Fujian clade. (TIF) [file pone.0018664.s002.tif]

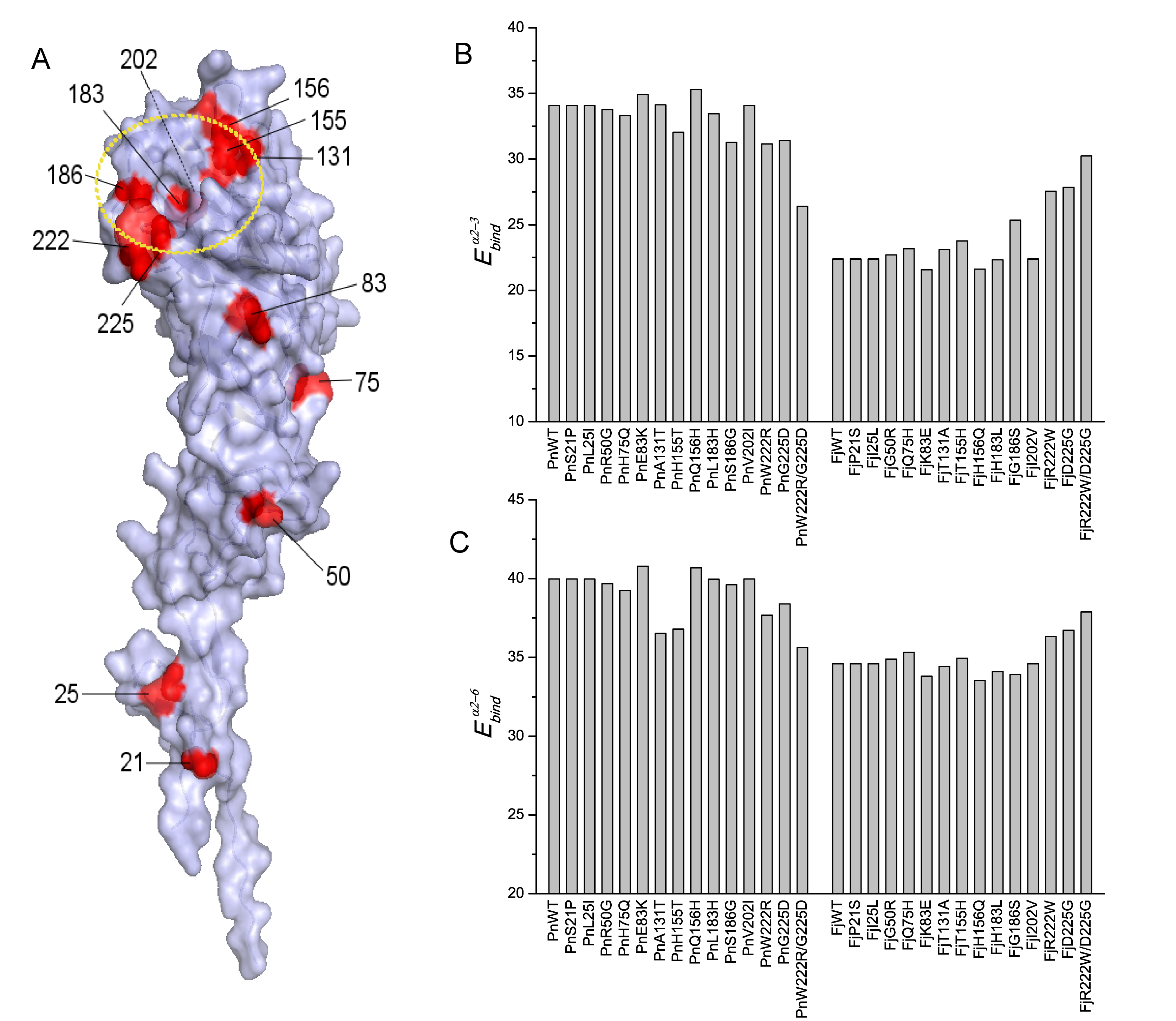

Supplement: Figure S3 — Computational identification of amino acid residues critical for altered receptor-binding specificity of Fujian-like viruses, related to Figure 2 . A: 13 different residues (red) between Panama and Fujian viruses on the structure of HA1. The binding region is highlighted by a yellow circle. B and C, Comparison of calculated binding strength of wildtype Panama (Pn) and Fujian (Fj) HAs to avian (α2–3) (b) and human (α2–6) (c) receptor analogs with specific amino acid changes in the HAs. To pinpoint the molecular changes responsible for the receptor specificity changes in A/Fujian/411/02, we modeled the effect on receptor binding of each mutation at all thirteen sites that differed between A/Panama/2007/99 and A/Fujian/411/02 viruses. For binding to the α2–3 sialic acid receptor (B), most single amino acid changes on the A/Panama/2007/99 HA did not have much effect except for changes at four residues, 155, 186, 222 and 225, which resulted in significant decreases in binding strength compared to the wild type. When positions 222 and 225 were changed simultaneously, the binding strength was further decreased. For single amino acid changes on the A/Fujian/411/02 HA backbone, only two changes at residues 222 and 225 resulted in a significant increase in binding strength compared to the wildtype. Similarly, simultaneous changes at positions 222 and 225 resulted in further decrease in binding strength. For binding to the α2–6 sialic acid receptor (C), residue 222 and 225 stood out again in that change at this position in HA of A/Panama/2007/99 and A/Fujian/411/02 viruses exhibited the reciprocal effect. These calculations suggest that the mutations at residues 222 and 225, which occurred at the beginning of the 2000–2001 flu season, played the most important role in mediating receptor binding alterations in the Fujian-like viruses. (TIF) [file pone.0018664.s003.tif]

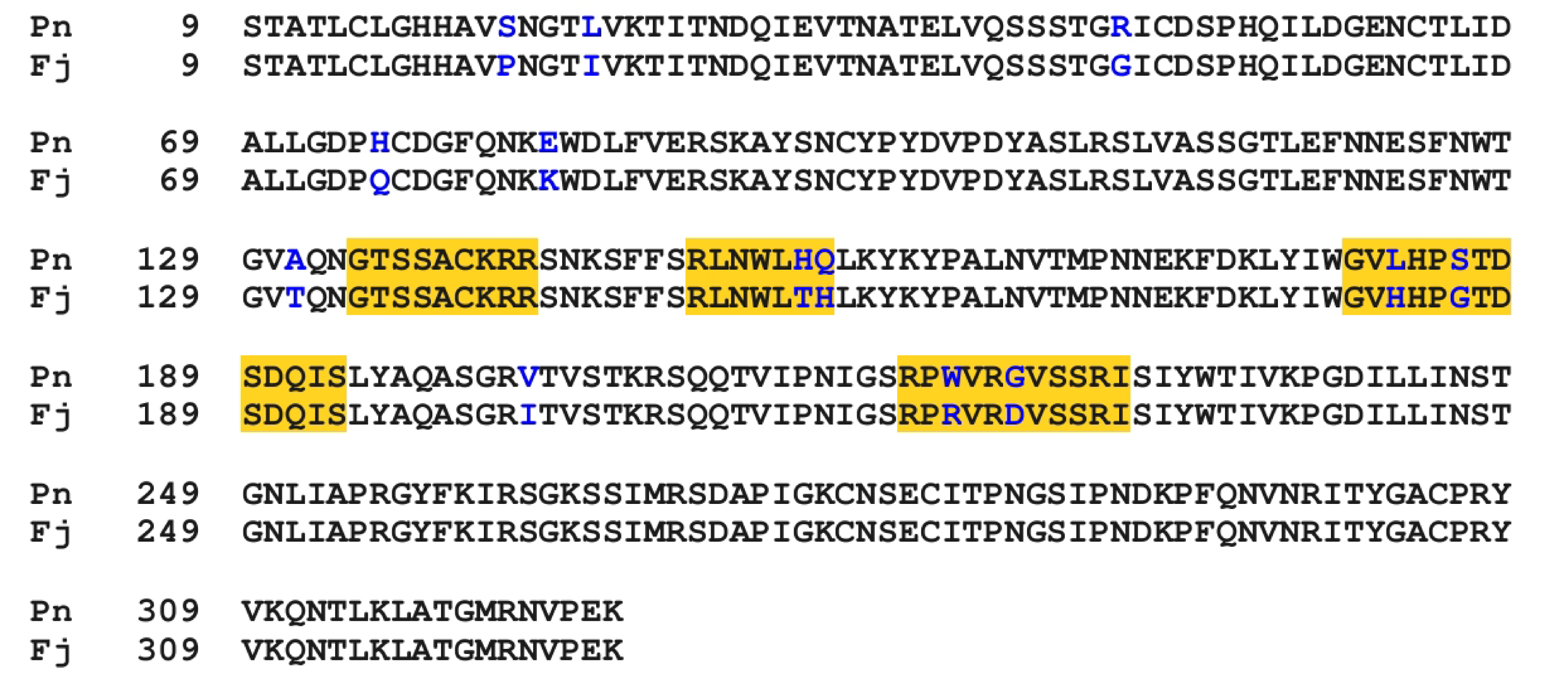

Supplement: Figure S4 — HA1 sequence alignment of Panama and Fujian virus. The 13 different residues are highlighted by the blue color. Receptor binding region comprises the 130-loop (134–142, H3 numbering), 150-loop (150–156), 190-helix (181–193) and 220-loop (220–230) (Yellow background). (TIF) [file pone.0018664.s004.tif]

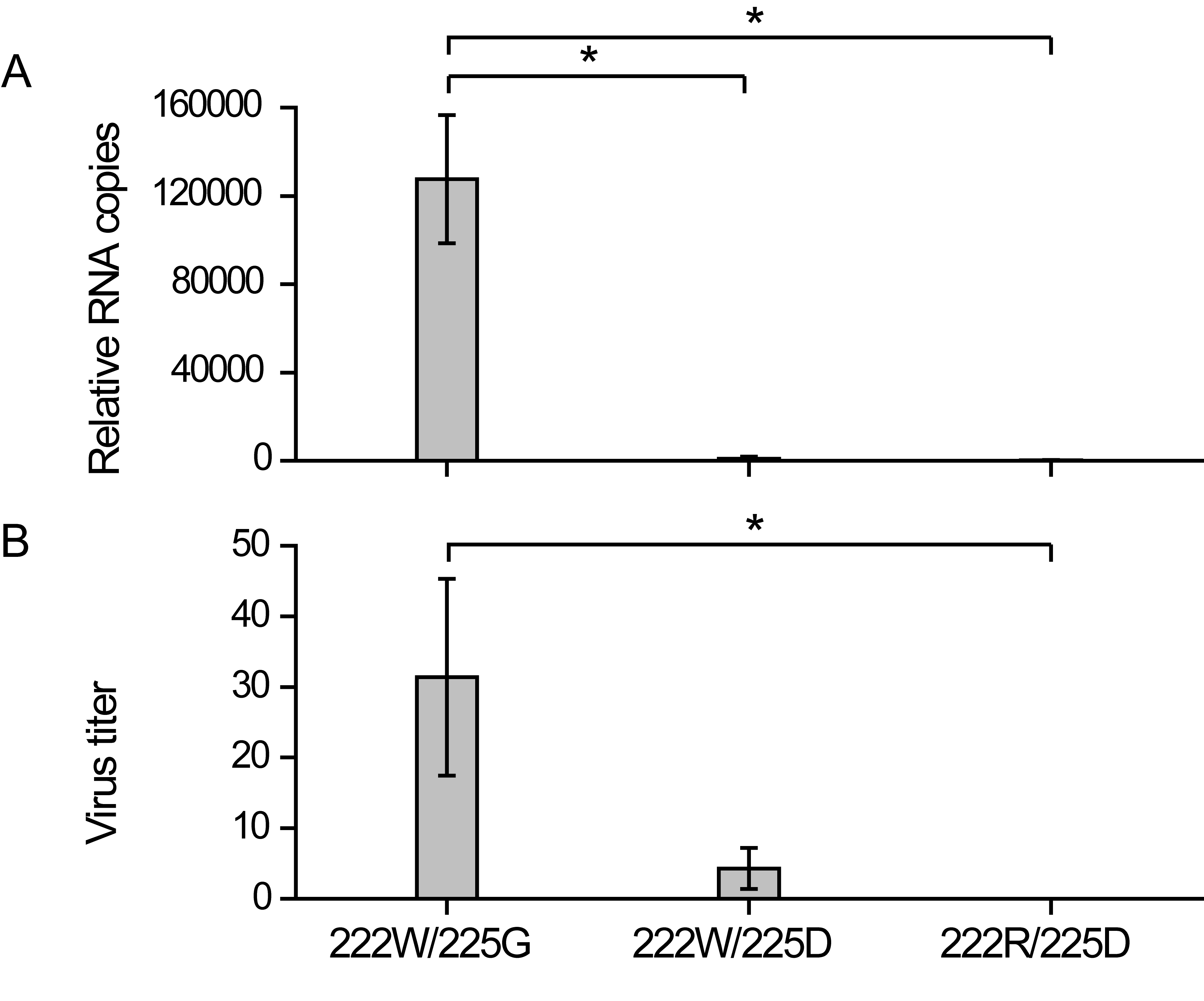

Supplement: Figure S5 — Comparison of growth of the Panama virus (222W/225G) and its variants 222W/225D and 222R/225D in embryonated chicken eggs, related to Figure 3 . A: Quantification by quantitative RT-PCR. The relative RNA copy is the ratio of the RNA copies in the embryonated chicken eggs after viral infection of 44 hours to those in the embryonated chicken eggs infected with same amount of viruses but kept frozen for 44 hours. (*p = 0.0009). B: Quantification by HA assay. The values of HA titers are average of either seven 222W/225G isolates, two 222W/225D isolates, or five 222R/225D isolates. (*p = 0.057) (TIF) [file pone.0018664.s005.tif]
