# Supplementary material for: Rapid Estimation of Binding Activity of Influenza Virus Hemagglutinin to Human and Avian Receptors
Source: PLoS One. 2011 Apr 13;6(4):e18664. doi: 10.1371/journal.pone.0018664 (PMC3076431; doi:10.1371/journal.pone.0018664)
Supplement: Table S1 — Comparison with popular scoring functions on PDBbind database. (DOC) [file pone.0018664.s006.doc]

**Table S1. Comparison with popular scoring functions on PDBbind database[5,6]**

| Scoring function | Pearson’s correlation coefficient | Standard deviation |
| --- | --- | --- |
| Ours | 0.510 | 1.86 |
| X-Score::HMScore | 0.566 | 1.82 |
| X-Score::HPScore | 0.514 | 1.89 |
| X-Score::HSScore | 0.506 | 1.9 |
| DrugScore::Pair | 0.473 | 1.94 |
| DrugScore::Surf | 0.463 | 1.95 |
| DrugScore::Pair/Surf | 0.476 | 1.94 |
| Sybyl::D-Score | 0.322 | 2.09 |
| Sybyl::PMF-Score | 0.147 | 2.16 |
| Sybyl::G-Score | 0.443 | 1.98 |
| Sybyl::ChemScore | 0.499 | 1.91 |
| Sybyl::F-Score | 0.141 | 2.19 |
| Cerius2::LigScore | 0.406 | 2 |
| Cerius2::PLP1 | 0.458 | 1.96 |
| Cerius2::PLP2 | 0.455 | 1.96 |
| Cerius2::PMF | 0.253 | 2.13 |
| Cerius2::LUDI1 | 0.334 | 2.08 |
| Cerius2::LUDI2 | 0.379 | 2.04 |
| Cerius2::LUDI3 | 0.331 | 2.08 |
| GOLD::GoldScore | 0.285 | 2.16 |
| GOLD::GoldScore_opt | 0.365 | 2.06 |
| GOLD::ChemScore | 0.423 | 2 |
| GOLD::ChemScore_opt | 0.449 | 1.96 |
| HINT | 0.33 | 2.08 |

Note: The above data of other scoring functions were obtained from the work of Wang et al. [7]
