# Supplementary material for: Rapid Estimation of Binding Activity of Influenza Virus Hemagglutinin to Human and Avian Receptors
Source: PLoS One. 2011 Apr 13;6(4):e18664. doi: 10.1371/journal.pone.0018664 (PMC3076431; doi:10.1371/journal.pone.0018664)
Supplement: Table S2 — Training data for the scoring function. (DOC) [file pone.0018664.s007.doc]

**Table S2.** Training data for the scoring function

| Virus① | $K_{ass}^{\alpha2-3}$($\mu M^{-1}$) | $K_{ass}^{\alpha2-6}$($\mu M^{-1}$) | $\log\left(\frac{K_{ass}^{\alpha2-6}}{K_{ass}^{\alpha2-3}}\right)$➁ |
| --- | --- | --- | --- |
| A/Duck/Hokkaido/10/85 | 60 | 3 | -1.30 |
| A/Duck/Hokkaido/9/85 | 60 | 3 | -1.30 |
| A/Duck/Hokkaido/7/82 | 60 | 2 | -1.48 |
| A/Duck/Hokkaido/33/80 | 60 | 3 | -1.30 |
| A/Duck/Hokkaido/8/80 | 20 | 1 | -1.30 |
| A/Duck/Ukraine/1/63 | 25 | 1.5 | -1.23 |
| A/Mallard/NY/6874/78 | 30 | 1.5 | -1.30 |
| A/Duck/Memphis/928/74 | 70 | <0.5 | -2.15 |
| A/Aichi/2/68 | 2 | 10 | 0.70 |
| A/Memphis/102/72 | 3 | 20 | 0.82 |
| A/Los Angeles/2/87 | 2 | 30 | 1.15 |
| A/Shanghai/11/89 | 2 | 20 | 1.00 |
| A/Turkey/MN/1661/81 | 60 | 1 | -1.78 |
| A/Mallard/TN/11464/85 | 60 | 1 | -1.78 |
| A/Duck/Alberta/35/76 | 70 | 1 | -1.85 |
| A/Duck/Bavaria/1/77 | 70 | 1 | -1.85 |
| A/Duck/Australia/749/80 | 45 | 1 | -1.65 |
| A/Swine/Netherlands/3/80 | 45 | 10 | -0.65 |
| A/Swine/Netherlands/12/85 | 30 | 8 | -0.58 |
| A/Swine/Italy/671/87 | 9 | 15 | 0.22 |
| A/Swine/Hokkaido/2/81 | 4 | 14 | 0.52 |

1. Experimental data is from the work of Matrosovich *et al.* [8].Virus sequences which did not exist on the NCBI's Influenza Virus Resource [2] were ignored.

➁ As the Matrosovich, M., A. Tuzikov, *et al.* pointed out that the relative affinity was much more reliable, we used the relative affinity value of $\log\left(\frac{K_{ass}^{\alpha2-6}}{K_{ass}^{\alpha2-3}}\right)$ as the training data.
