# Supplementary material for: Rapid Estimation of Binding Activity of Influenza Virus Hemagglutinin to Human and Avian Receptors
Source: PLoS One. 2011 Apr 13;6(4):e18664. doi: 10.1371/journal.pone.0018664 (PMC3076431; doi:10.1371/journal.pone.0018664)
Supplement: Table S3 — Validation of the computational method using well characterized mutations. (DOC) [file pone.0018664.s008.doc]

**Table S3. Validation of the computational method using well characterized mutations**.

| HA | Ref | Mutation | Binding to avian receptor analog | | | Binding to human receptor analog | | |
| --- | --- | --- | --- | --- | --- | --- | --- | --- |
|  |  |  | $\Delta E_{bind}$ | Predicted activity | Observed activity | $\Delta E_{bind}$ | Predicted activity | Observed activity |
| A/South Carolina/1/1918 | [9] | D225G | 1.2 | ↑ | ↑ | 1.1 | ↑ | - |
|  |  | D225G D190E | 2.6 | ↑ | ↑ | -1.2 | ↓ | ↓ |
| A/duck/Ukraine/1/63 | [10] | Q226L | -1.8 | ↓ | ↓ | 0.0 | - | ↑ |
| A/Udorn/307/72 | [11] | L226Q | 1.1 | ↑ | ↑ | 0.8 | - | NA |
|  |  | L226Q S228G | 1.9 | ↑ | ↑ | 2.6 | ↑ | NA |
| A/Paris/906/97 | [12] | S193R | 1.2 | ↑ | ↑ | 1.9 | ↑ | ↑ |
| A/Vietnam/1194/04 | [13] | G139R | 0.8 | - | - | 0.9 | - | - |
|  |  | N182K | -0.5 | - | -① | 1.7 | ↑ | ↑ |
|  |  | G139R N182K | 0.3 | - | - | 2.6 | ↑ | ↑ |
|  |  | Q192R | 0.4 | - | -① | 1.4 | ↑ | ↑ |
|  |  | S223N | -0.3 | - | -① | 1.0 | ↑ | - |
|  |  | Q192R S223N | 0.4 | - | -① | 2.4 | ↑ | ↑ |

①Binding strength exhibits a certain degree of decrease according to the original paper; however, we do not interpret them to be critical mutations.

The symbols ↑, ↓ and – denote increased, decreased and unchanged binding strength, respectively. NA indicates data not available. For mutations, $\Delta E_{bind}$ with a value ≥1.0,≤ -1.0, between -1.0 and 1.0 indicates, respectively, increased (↑), decreased (↓) and unchanged (–) binding strength.

The numbering of mutation sites follows the relative literatures for the convenience of comparable reading. In case of A/Vietnam/1194/04, 139, 182, 192 and 223 is the same residues of 143, 186, 196 and 227 in H3 numbering.
