# Supplementary material for: Rapid Estimation of Binding Activity of Influenza Virus Hemagglutinin to Human and Avian Receptors
Source: PLoS One. 2011 Apr 13;6(4):e18664. doi: 10.1371/journal.pone.0018664 (PMC3076431; doi:10.1371/journal.pone.0018664)
Supplement: Methods S1 — Supporting methods. (DOC) [file pone.0018664.s009.doc]

**Supporting Methods S1**

**Rapid Estimation of Binding Activity of Influenza Virus Hemagglutinin to Human and Avian Receptors**

Yang Cao, Xiaoying Koh, Libo Dong, Xiangjun Du, Aiping Wu, Xilai Ding, Hongyu Deng, Yuelong Shu, Jianzhu Chen, Taijiao Jiang.

*Structure Models:* Crystal structures were prepared from Protein Data Bank (PDB) [1]. Complex structures of HA binding with the α2-3/6 receptor analogs (PDB ID: 1RVX, 1RVZ, 1MQM, 1MQN, 1JSN, 1JSO, 1JSH, 1JSI) were used to parameterize the scoring function for side chain repacking. Complete LSTa/c were extracted from the crystal structures (PDB ID：2RFT，1RVT，1JSI).

*Selection of HA Sequences of human/avian H1, H3 and H5 viruses:* 1188, 6451 and 271 human H1N1, H3N2 and H5N1 HA sequences were obtained from NCBI's Influenza Virus Resource [2]. They were processed by removing those with missing residues at the receptor binding region and keeping only one sequence from each group of sequences with identical residues at the receptor binding site (residue 111-265 H3 numbering [3]). As the number of H1N1 and H3N2 sequences was still too large, remaining sequences were clustered according to the number of residue differences in the receptor binding sites. No more than 11 sequences per year with different geographical information in each cluster were randomly selected out. In this way, 193, 360 and 144 human H1, H3 and H5 HA sequences were selected. Similarly 77, 240 and 1664 avian H1N1, H3 and H5N1 HA sequences were obtained from NCBI's Influenza Virus Resource [2]. The sequences that are identical in receptor binding site are removed. Then 10 more avian H3 HA1 sequences were removed because these viruses are transmitted from swine to turkeys [4]. H5N1 HA sequences were processed in a similar way as human H1N1 and H3N2 sequences. Finally, 75, 152 and 686 avian H1, H3 and H5 HA sequences were selected. For each subtype, the sequences are highly similar (over 70% of sequence identity) to the sequence of the crystal structure used as template in the homology modeling of their structures.

**Scoring function for side chain repacking**

To optimize the conformations of side chains of receptor binding site on HA when binding with receptor analogs, a scoring function for side chain repacking was developed:

$E=s_{1}E_{lib}+s_{2}E_{vdW}+s_{3}E_{sb}+s_{4}E_{solv}$ (7)

Where $E_{lib}$, $E_{vdW}$, $E_{sb}$, $E_{solv}$ are Dunbrack rotamer library term [14], van der Waals term, salt bridge interaction term and simple solvation energy term. Residues are represented by their heavy atoms.

The van der Waals term consists of simple repulsion term and attraction term as follow:

$E_{vdW}=\left\{\begin{matrix} \sum_{i<j} 1.0 for r_{ij}\leq0.75R_{ij} \\ \sum_{i<j} 4.0(1-r_{ij}/R_{ij}) for 0.75R_{ij}<r_{ij}\leq R_{ij} \\ \sum_{i<j} -2.5(r_{ij}/R_{ij})^{2}+6.0(r_{ij}/R_{ij})-3.5 for R_{ij}<r_{ij}<1.4R_{ij} \\ 0 for r_{ij}\geq1.4R_{ij} \end{matrix}\right.$ (8)

where $r_{ij}$ is the interatomic distance for atoms $i$ and $j$, and $R_{ij}$ is the sum of their van der Waals radii.

The salt bridge term is used to capture the energy of salt bridge interactions:

$E_{sb}=\left\{\begin{matrix} \sum_{i<j} 332 q_{i}q_{j} for r\leq3.5 \\ \sum332q_{i}q_{j}(1-(r_{ij}-3.5)^{2}/4) for 3.5<r<4.5 \\ 0 \text{ } for r\geq4.5 \end{matrix}\right.$ (9)

Where $r_{ij}$ is the interatomic distance of point charges $i$ and $j$, from different residues, on which positive unit charges were positioned at NZ of lysine, NH1 or NH2 of arginine, ND1 or NE2 of histidine, and negative unit charges at OD1 or OD2 of aspartate, OE1 or OE2 of glutamate. The unit charges were then scaled by accessible surface areas ($S_{asa}$) of the charged atoms:

$q=\left\{\begin{matrix} 1.0 for S_{asa}\leq S_{ref} \\ 1.0\frac{S_{ref}}{S_{asa}} for\text{ } S_{asa}>S_{ref} \end{matrix}\right.$ (10)

Where $S_{ref}$ is set to be 1/12 of the charged atom’s surface area. The precise positions of the above charges with 2 choices are determined by searching the minimum salt bridge term with a depth first search program.

The simple solvation term $E_{solv}$ is based on the atomic solvation parameters of Eisenberg and McLanchlan [15] with a surface area calculation algorithm of Sharke and Rupley [16].

To accurately model the conformation of side chains of HA in binding with receptor analogs, the weights of the scoring function for side chain repacking were parameterized on crystal structures of HA with the α2-3/6 receptor analogs ($s_{1}=0.2$,$s_{2}=1.0$,$s_{3}=0.1$,$s_{3}=3.0$).

**Sequencing and sequence analysis**

HA sequences used in this analysis were generated at the Chinese Influenza Center as part of an ongoing routine genetic analysis of HA genes of variant and typical influenza field strains. Viruses were propagated at a low multiplicity of infection in embryonated chicken eggs or Madin-Darby canine kidney (MDCK) cells. Viral RNA was extracted from cleared supernatant using the Qiagen RNeasy Mini Kit. HA1 nucleotide fragments were amplified by reverse transcription-PCR using primer 5'-AGCAAAAGCAGG-3′. The PCR products were purified and subjected to sequencing using primers 5'-AGCAAAAGCAGGGGATAATT-3' and 5'-CCTGCGATTGCGCCGAAT-3′. Phylogenetic analyses of HA sequences were performed using the maximum likelihood method available in the Phylogeny Inference Package [17](PHYLIP). Default parameters were used.

**Hemadsorption glycan-binding assay**

The hemadsorption glycan-binding assay protocol was modified from Glaser *et al.* [9]. 10% chicken red blood cells (Charles River Laboratories) were incubated with 50 mU neuraminidase (Roche) at 37°C for 1 h to remove all of the sialic acid. Complete removal of sialic acid was tested by the hemagglutination assay using the A/PuertoRico/8/34 virus, and unmodified chicken red blood cells were used as controls. Resialylation of chicken red blood cells was performed by incubating 100 μl chicken red blood cells with either 0.5 mU α2-3-(N)-sialyltransferease (Calbiochem) or 1.0 mU α2-6-(N)-sialyltransferase (a gift from Dr. James Paulson, Scripps), 1.5 mM CMP-sialic acid at 37°C for 1 h. 293T human embryonic kidney cells in 6-well plates were transfected with 2 μg of HA in pcDNA3.1 vector using Mirus transfection reagent (Mirus Bio, LLC). 48 hrs later, the transfected 293T cells were treated with 5 mU neuraminidase for 30 min to remove sialic acids on the expressed HA that may interfere with glycan-binding, and washed three times in PBS, pH 7.4. 2ml of 0.5% resialylated chicken red blood cells were incubated with the 293T cells at 4°C for 30 min, and carefully washed three times in PBS to remove unbound chicken red blood cells. The bound chicken red blood cells were lysed in 375 μl of lysis buffer (20 mM Tris-HCl, 177mM NH_4_Cl, pH 7.5) for 30 min at room temperature. The amount of bound chicken red blood cells was quantified by measuring the absorbance of released heme groups at 540 nm. The negative control which consists of 293T cells not transfected with HA but expressed GFP shows consistently low, background signals as compared to cells expressing HA. It implies that 1) chicken red blood cells have been successfully resialylated, and 2) resialylated chicken red blood cells discriminate between HA and non-HA expressing 293T cells.

**Propagation and titration of viruses in chicken embryonated eggs**

Based on the particular amino acid residues at sites 222 and 225 of HA, the Panama clade of viruses isolated in China during 2000-2002 were divided into three groups: 222W/225G for viruses with tryptophan (W) and glycine (G) at positions 222 and 225, respectively; 222W/225D for viruses with tryptophan (W) and aspartic acid (D) at positions 222 and 225, respectively; and 222R/225D for viruses with arginine (R) and aspartic acid (D) at positions 222 and 225, respectively. Seven isolates of 222W/225G type, 2 isolates of 222W/225D type and 5 isolates of 222R/225D type were tested for their ability to grow in embryonated chicken eggs. Embryonated chicken eggs (the amniotic and allantoic cavity of 10-day-old eggs) were infected with equal amount of viruses. Forty-four hours later, the replication of the viruses was quantified by either by hemagglutination assays or real-time RT-PCR (reverse transcription polymerase chain reaction). The hemagglutination assays were performed with 0.5% suspension of Cavia porcellus red blood cells. The replicative abilities of CE grown viruses were also measured by RT-PCR using primer specific for the gene encoding the matrix protein M1. Viral RNA was extracted from amniotic fluid by using a Qiagen RNeasy mini kit according to the manufacturer’s directions (Qiagen, Hilden, Germany). H3 specific primer and probe sets for conserved region in M gene were designed ( Forward primer: 5'-GACCRATCCTGTCACCTCTGAC-3', reverse primer: 5’-GGGCAITYTGGACAAAKCGTCTACG-3’,probe:5'-TGCAGTCCTCGCTCACTGGGCACG-3'). The hydrolysis probe was labeled with 6-carboxyfluorescein (FAM) at the 5’ end, and 6-carboxytetramethylrhodamine as a quencher dye at the 3’ end. The QuantiTect™ Probc RT-PCR kit was used. The primers and probes were used at a final concentration of 0.8μmol/L and 0.2μmol/L separately. The RT-PCR took place in a final volume of 25μl using a Applied Biosystems™ real-time PCR systems(7000) and the data was analyzed by software MaxPro. The following protocol was used for all primer/probe sets: 30 min at 50°C and 10 min at 95°C, followed by 45 cycles at 95°C for 15 s; 55°C for 30 s; 72°C for 30 s. Fluorescence data (FAM) should be collected during the 55°C incubation step. An avian influenza virus was taken as positive control, and the embryonated chicken eggs that were injected with same amount of viruses and then kept frozen were taken as references prior to viral replication. All samples exhibited reaction curves that cross the threshold line at or before 35 cycles, indicating the specimen is of acceptable quality.

**References**

1. Berman H, Westbrook J, Feng Z, Gilliland G, Bhat TN, et al. (2000) The Protein Data Bank. Nucl Acids Res 28: 235-242.

2. Bao Y, Bolotov P, Dernovoy D, Kiryutin B, Zaslavsky L, et al. (2008) The influenza virus resource at the National Center for Biotechnology Information. Journal of virology 82: 596-601.

3. Gamblin SJ, Haire LF, Russell RJ, Stevens DJ, Xiao B, et al. (2004) The Structure and Receptor Binding Properties of the 1918 Influenza Hemagglutinin. Science 303: 1838-1842.

4. Choi YK, Lee JH, Erickson G, Goyal SM, Joo HS, et al. (2004) H3N2 influenza virus transmission from swine to turkeys, United States. Emerging infectious diseases 10: 2156-2160.

5. Wang R, Fang X, Lu Y, Wang S (2004) The PDBbind Database: Collection of Binding Affinities for Protein−Ligand Complexes with Known Three-Dimensional Structures. Journal of Medicinal Chemistry 47: 2977-2980.

6. Wang R, Fang X, Lu Y, Yang C-Y, Wang S (2005) The PDBbind Database: Methodologies and Updates. Journal of Medicinal Chemistry 48: 4111-4119.

7. Wang R, Lu Y, Fang X, Wang S (2004) An Extensive Test of 14 Scoring Functions Using the PDBbind Refined Set of 800 Protein−Ligand Complexes. Journal of Chemical Information and Computer Sciences 44: 2114-2125.

8. Matrosovich M, Tuzikov A, Bovin N, Gambaryan A, Klimov A, et al. (2000) Early alterations of the receptor-binding properties of H1, H2, and H3 avian influenza virus hemagglutinins after their introduction into mammals. Journal of virology 74: 8502-8512.

9. Glaser L, Stevens J, Zamarin D, Wilson I, García-Sastre A, et al. (2005) A single amino acid substitution in 1918 influenza virus hemagglutinin changes receptor binding specificity. Journal of virology 79: 11533-11536.

10. Rogers GN, Daniels RS, Skehel JJ, Wiley DC, Wang XF, et al. (1985) Host-mediated selection of influenza virus receptor variants. Sialic acid-alpha 2,6Gal-specific clones of A/duck/Ukraine/1/63 revert to sialic acid-alpha 2,3Gal-specific wild type in ovo. The Journal of biological chemistry 260: 7362-7367.

11. Vines A, Wells K, Matrosovich M, Castrucci MR, Ito T, et al. (1998) The role of influenza A virus hemagglutinin residues 226 and 228 in receptor specificity and host range restriction. Journal of virology 72: 7626-7631.

12. Medeiros R, Naffakh N, Manuguerra JC, van der Werf S (2004) Binding of the hemagglutinin from human or equine influenza H3 viruses to the receptor is altered by substitutions at residue 193. Archives of Virology 149: 1663-1671.

13. Yamada S, Suzuki Y, Suzuki T, Le M, Nidom C, et al. (2006) Haemagglutinin mutations responsible for the binding of H5N1 influenza A viruses to human-type receptors. Nature 444: 378-382.

14. Dunbrack R, Cohen F (1997) Bayesian statistical analysis of protein side-chain rotamer preferences. Protein Science 6: 1661-1681.

15. Eisenberg D, McLachlan AD (1986) Solvation energy in protein folding and binding. Nature 319: 199-203.

16. Shrake A, Rupley JA (1973) Environment and exposure to solvent of protein atoms. Lysozyme and insulin. Journal of Molecular Biology 79: 351-371.

17. Guindon S, Gascuel O (2003) A simple, fast, and accurate algorithm to estimate large phylogenies by maximum likelihood. Systematic biology 52: 696-704.
